# Impact of Left Atrial Appendage Closure Combined with Catheter Ablation on Endocrine and Mechanical Cardiac Function in Patients with Atrial Fibrillation

**DOI:** 10.1155/2022/3932912

**Published:** 2022-03-19

**Authors:** Jing Yang, Ling You, Mingqing Liu, Guangming Zhang, Liang Feng, Yue Liu, Xue Geng, Jinglan Wu, Ruiqin Xie

**Affiliations:** ^1^Division of Cardiology, The Second Hospital of Hebei Medical University, Shijiazhuang, Hebei, China; ^2^Cangzhou Medical College, Cangzhou, Hebei, China

## Abstract

**Background:**

The combined procedure of left atrial appendage closure (LAAC) and catheter ablation (CA) is a safe and feasible therapy to treat patients with atrial fibrillation (AF). However, the effect of the combined procedure on cardiac function remains unclear. This study aimed to investigate the changes in endocrine and mechanical function of the heart following the combined procedure.

**Methods:**

This retrospective study included 62 consecutive patients who underwent the combined procedure of AF ablation and WATCHMAN LAAC and 62 sex and age-matched patients who only received AF ablation. During follow-up, patients were examined for brain natriuretic peptide (BNP) levels to represent endocrine cardiac function. Mechanical cardiac function was assessed during echocardiographic examination by means of the LA ejection fraction, LA strain (*Ƹ*), and LA strain rate (SR).

**Results:**

(1) The BNP levels decreased acutely after the procedure, rose at day 3 postoperation, but trended downwards at 3 months postoperation in both groups. No significant difference was observed between the two groups. (2) LA ejection fraction, LA *Ƹ*, and SR exhibited a continuous upward trend over a 3-month follow-up in both groups. There was no significant difference in LA ejection fractions, SRe (the parameter of LA conduit function), and SRa (the parameter of LA booster pump function) between the two groups. However, the *Ƹ* and SRs (the parameters of LA reservoir function) improved in patients treated with CA alone.

**Conclusions:**

The combined procedure of LAAC and CA significantly improved the endocrine and mechanical function of the heart. Compared to simple CA, based on CA with LAAC intervention, it does not significantly change LA endocrine function but may lead to a decline in the LA reservoir function.

## 1. Background

Atrial fibrillation (AF) is the most commonly diagnosed arrhythmia worldwide and has been recognized as a major cause of ischemic stroke. In recent years, drug-refractory AF has been increasingly treated with catheter ablation (CA). However, the long-term outcomes remain uncertain due to significant recurrence over time, especially in patients with persistent AF and hypertension [1]. The left atrial appendage (LAA) is the source of thrombi that cause strokes in most patients with nonvalvular atrial fibrillation (NVAF). Accordingly, left atrial appendage closure (LAAC) has been used as a nonpharmacologic alternative for stroke prophylaxis in NVAF patients [2, 3]. It can be performed as a stand-alone procedure or in combination with CA, which enables sinus rhythm (SR) control and stroke prevention in one single process. Recent studies have shown that the combination of LAAC with CA is safe and feasible for AF patients, no matter the energy source for pulmonary vein isolation [4, 5].

The LAA was originally considered as a vestigial structure. Emerging evidence has highlighted the importance of LAA in neurohormonal regulation and cardiac hemodynamics. Owing to its hormone-producing and contractile function, the LAA is essential for fluid and electrolyte balance in the human body. The occlusion of LAA is still somewhat controversial [6]. Therefore, it is of great importance to develop novel strategies for LAA management. CA has been shown to improve LA function and regulate the expression of neuroendocrine markers [7]. As an approved procedure, the effect of LAAC on cardiac function has been widely discussed. However, no published literature has reported the therapeutic potential of the one-stop hybrid procedure combining CA and LAAC in neurohormonal regulation and cardiac hemodynamics. Furthermore, the effect of LAAC on cardiac function following CA has not been fully studied. In this study, we explored the changes in cardiac function, including BNP and echo parameters, in AF patients following the combined procedure and simple CA. By comparing the changes in endocrine and mechanical cardiac function between the two groups, we further investigated whether LAAC would exert beneficial or detrimental effects on CA. These results may provide insights to optimize the LAAC procedure from the aspect of cardiac function.

## 2. Methods

### 2.1. Study Population

**Figure 1:**
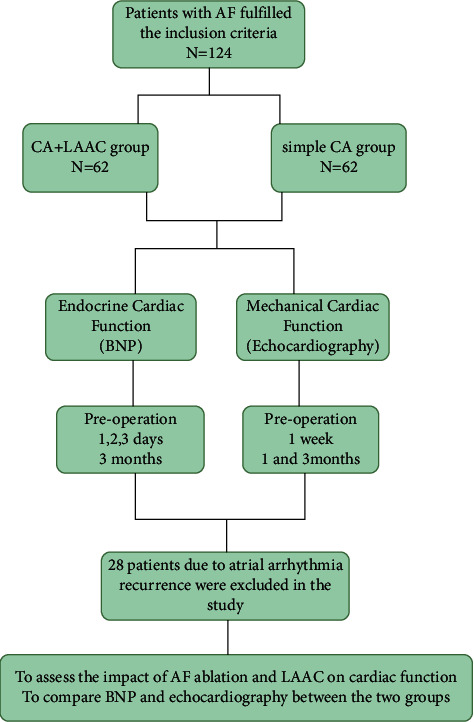
Flowchart of study procedure. LA, left atrial; BNP, brain natriuretic peptide; LAAC, left atrial appendage closure; CA, catheter ablation; AF, atrial fibrillation.

In this single-center, retrospective study, 62 consecutive patients who underwent the combined procedure of AF ablation and WATCHMAN LAAC in our center between July 2017 and March 2019 were recruited (Group 1). Sixty-two sex- and age-matched patients who only received AF ablation were also enrolled (Group 2). The flowchart of the recruitment process is shown in Figure 1. The inclusion criteria were as follows: (1) age 18 years or older; (2) symptomatic NVAF refractory to antiarrhythmic drugs; (3) one or more CHA2DS2-VASc risk factors (age ≥75 years, hypertension, diabetes, heart failure or left ventricular systolic dysfunction, prior transient ischemic attack, or stroke); (4) high bleeding risk or previous major bleeding event in anticoagulation therapy; (5) preferred surgical treatment as an alternative to long-term oral anticoagulants. Patients with LA thrombus, significant valvular heart disease, an enlarged LA (≥55 mm), and additional ablation lines or CFAE ablation strategy were excluded. The study protocol was approved by the ethics committee of the Second Hospital of Hebei Medical University, in agreement with the ethical guidelines of the 1975 Declaration of Helsinki. Written consent was obtained from all patients.

### 2.2. CA Procedure

Transesophageal echocardiography and cardiac computed tomography were performed to exclude patients with LAA thrombus and to evaluate the dimension and depth of the appendage prior to the operation. CA was performed under conscious sedation and local anesthesia. Pulmonary vein isolation was achieved in all cases using radiofrequency. In brief, the ablation catheter (Thermocool SMART-TOUCH, Biosense Webster) was inserted into the LA for radiofrequency ablation under the 3D electroanatomical mapping system (Carto 3, Biosense Webster). The mapping catheter (Lasso® NAV Eco, Biosense Webster) was used to record pulmonary vein potentials before, during, and after ablation. The ablation strategy was confirmed by electrical isolation through circumferential ablation around both left and right pulmonary veins. No additional ablation lines or CFAE ablation were performed in this study. Ibutilide was used when patients had AF during surgery. For patients who did not restore SR after ablation, additional cardioversion was performed to terminate AF.

### 2.3. LAAC Procedure

**Figure 2:**
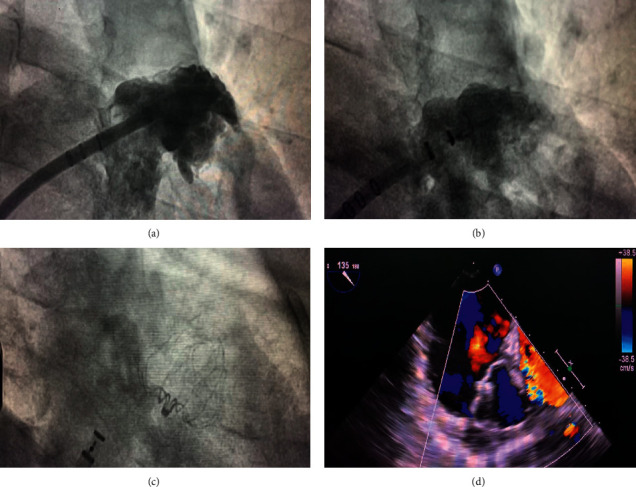
Left atrial appendage closure after catheter ablation with WATCHMAN device. (a) Left atrial appendage angiograph. (b, c) Fluoroscopic view after delivery of the WATCHMAN device; (d) peridevice leakage and compression were detected by TEE postimplantation.

After the CA procedure, LAAC was carried out using an occluder device (WATCHMAN, Boston Scientific, Marlborough, MA, USA). All patients undergoing epicardial LAAC procedures received up to 1,500 mL of 0.9% normal saline intravenous infusion to ensure the filling of the LAA. The process of LAAC implantation is shown in Figure 2. LAA angiography was performed to measure the width and depth of the ostium. A device with a size of 10–20% larger than the largest diameter of the LAA was recommended. The following criteria need to be fulfilled before the release of the device from the delivery catheter, including proper LAA position, no or minimal (<5 mm) residual lateral flow passing the device, and a tug test for stability.

### 2.4. Follow-Up

Patients were followed for three months and the echocardiography was performed at 1 week, 1 month, and 3 months after the treatment using an iE33 echocardiography system equipped with an X3-1 probe (Philips Medical Systems, Eindhoven, Netherlands). Patients were excluded if AF or atrial flutter was detected during follow-up.

LA volumes were measured by the single-plane Simpson's method through an apical four-chamber view. The maximum LA volume (LAV max) referred to the LA volume measured at the end-diastolic frame preceding mitral valve opening, whereas the minimum LA volume (LAV min) referred to the volume measured at the end-systolic frame preceding mitral valve closure. The LA ejection fraction (LAEF) was calculated using the formula: LAEF = [(LAV max − LAV min)/LAV max] × 100%.

**Figure 3:**
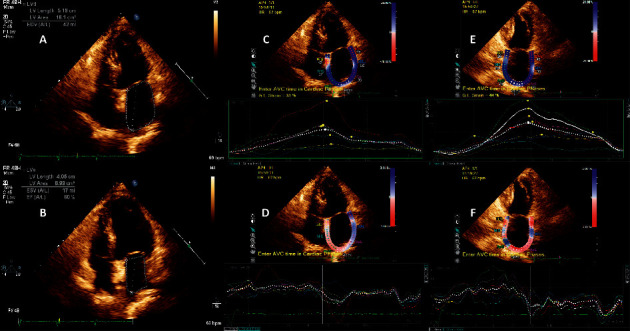
Cardiac function measured by echocardiography and speckle tracking echocardiography. (a, b) Measurement of maximum LA volume and minimum LA volume by the single-plane Simpson's method through an apical four-chamber view. (c, d) Atrial strain and strain rate measured in an apical four-chamber view. (e, f) Atrial strain and strain rate measured in an apical two-chamber view.

The longitudinal LA strain (*Ƹ*) and strain rate (SR) were also analyzed using speckle tracking echocardiography (STE), a two-dimensional cardiac performance analysis (QLAB software, Philips Medical Systems), as previously described [8]. The preoperative *Ƹ* and SR including SRs and SRe were measured under SR and AF. As SRa was only measured under SR, its postoperative changes were analyzed. The process of echocardiography and STE is shown in Figure 3.

Venous blood samples were obtained for BNP assessment at 1 day, 2 days, 3 days, and 3 months after the operation. All patients received anti-arrhythmic drugs and anticoagulation regimens after the operation for 3 months. The rest of the blood pressure medications were routinely continued, and no other routine medications were given. All patients underwent systematic transesophageal echocardiography (TEE) at 3-month follow-up. The 24-hour Holter was obtained at 1- and 3-months' follow-up to detect atrial arrhythmias.

### 2.5. Statistical Analysis

The data were analyzed using SPSS Statistics, version 20 (IBM, Armonk, NY). The Kolmogorov–Smirnov test was applied to determine whether the data followed a normal distribution. Normally distributed data were expressed as the mean ± standard deviation, while nonnormally distributed data were shown as medians with interquartile ranges. Counting data were expressed as percentages (%). Continuous data of different indices were assessed using the analysis of variance for repeated measures. The student's *t*-test (normality) or Mann–Whitney *U* test (nonnormality) methods were used to compare variables between the two groups. The Chi-square test was used to compare qualitative data. A *p* value of <0.05 was considered statistically significant.

## 3. Results

### 3.1. Baseline Data

A total of 124 patients were included in this study, and their characteristics at baseline are shown in Table 1. Of them, 62 were treated with CA and LAAC (Group 1) and the rest only received CA (Group 2). There was no significant difference in age or previous medical history between the two groups. Hypertension was the most common comorbidity (50% in Group 1 and 63% in Group 2, *p*=0.15), followed by coronary artery disease (42% in Group 1 and 29% in Group 2, *p*=0.13) and diabetes (15% in Group 1 and 15% in Group 2, *p*=1.00).

### 3.2. Endocrine Cardiac Functions

**Figure 4:**
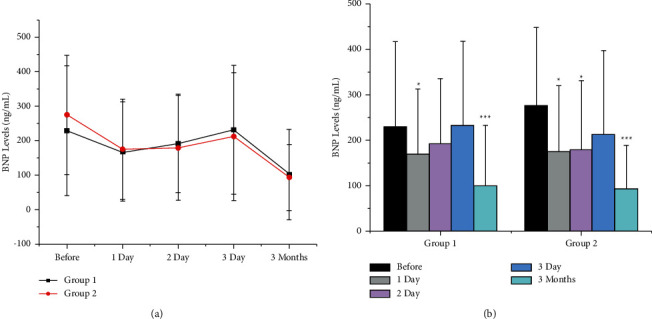
Changes in BNP levels over time. (a) Changes in BNP levels over time in both groups. There was no significant difference in BNP levels between the two groups. (b) The trend of changes in BNP levels in each group. Variables are expressed as the mean ± SD. BNP, brain natriuretic peptide; Group 1: LAAC combined with CA. Group 2: simple CA group. ^*∗*^*p* < 0.05; ^*∗∗∗*^*p* < 0.001 compared with the baseline level.

Out of 124 patients, 28 were excluded due to AF or atrial flutter, leaving 96 patients (47 in Group 1 and 49 in Group 2) for the analysis of BNP and echo parameters. Blood samples were collected at five time points for the assessment of BNP to evaluate the endocrine cardiac function (Figure 4). In patients treated with CA and LAAC, the BNP levels acutely decreased with the restoration of SR, rose until day 3 postoperation, and then trended downwards at 3 months postoperation. The same trend was observed for the BNP levels in the simple CA group. There was no significant difference concerning BNP levels between the two groups.

### 3.3. Mechanical Cardiac Functions

LA function parameters including LAEF, *Ƹ*, and LA SR exhibited a continuous upward trend over a 3-month follow-up in both groups. In Group 1, LAEF, LA strain, and LA SR (SRs and SRe) significantly increased in both the two-chamber and four-chamber views. SRa, the parameter of the LA booster pump function, significantly increased in the four-chamber but did not change in the two-chamber view. In Group 2, LAEF, LA strain, and LA SR (SRs and SRa) significantly increased in both the two-chamber and four-chamber views. SRe, the parameter of the LA conduit function, showed an increasing trend, but no statistical difference was found in both chambers. The correlational analysis is shown in Table 2.

**Figure 5:**
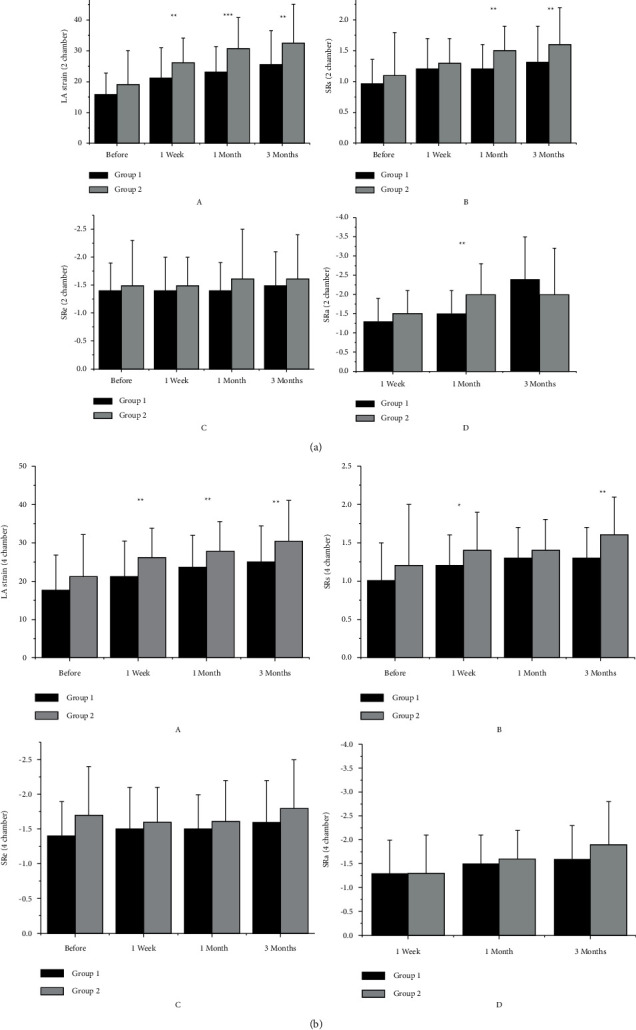
Comparison of strain indices between the two groups in both two-chamber (a) and four-chamber views (b). Group 1: LAAC combined with CA. Group 2: simple CA group. Variables are expressed as the mean ± SD. SR, strain rate. SRs, strain rate during ventricular systole; SRe, strain rate during early ventricular diastole. SRa, strain rate during atrial systole. ^*∗*^*p* < 0.05; ^*∗∗*^*p* < 0.05; ^*∗∗∗*^*p* < 0.001 vs. simple CA group.

There was no significant difference in LAEF, SRe, or SRa between the two groups. However, compared with patients who underwent the combined procedure, those in Group 2 showed improved *Ƹ* and SRs (the parameters of the LA reservoir function). The LA function assessed in both two- and four-chamber views in both groups is shown in Figure 5.

### 3.4. Clinical Outcomes

Pulmonary vein isolation was achieved in both groups, and SR was restored in all patients. During the 3-month follow-up, 15 patients (24.2%) in Group 1 and 13 patients (21.0%) in Group 2 had a recurrence of AF or atrial flutter (*p*=0.67). In the group treated with both CA and LAAC, complete device occlusions were achieved in all patients. One patient (1.6%) had pericardial effusion. No stroke or death was observed during the follow-up period. At 3 months postoperation, no dislocation event, thromboembolic event, or ischemic stroke was recorded. A device-related thrombus was observed in one patient undergoing TEE at 3-month follow-up. In Group 2, no procedural complications were observed at 3-month postoperation, with the exception of one case with cerebral hemorrhage. Periprocedural characteristics and postoperative complications are summarized in Table 3.

## 4. Discussion

Our study showed that the combined procedure of CA and LAAC significantly improved endocrine and mechanical cardiac function in AF patients, and CA, rather than LAAC, was responsible for most of the changes. Compared to simple CA, based on CA with LAAC intervention, it does not significantly change LA endocrine function, but may lead to a decline in the LA reservoir function.

### 4.1. Endocrine Cardiac Function

We found that the endocrine cardiac function, as indicated by the levels of BNP, improved 3 months after the hybrid procedure. This finding may be associated with significant restoration of mechanical atrial function after CA [9] and complete endothelialization of the LAAC device [10]. Moreover, the plasma BNP levels decreased acutely one day after the CA and LAAC operations, which might be attributed to the recovery of SR [9, 11, 12], recovered within 3 days, and returned to the baseline levels. Previous studies have reported a gradual increase in the levels of natriuretic peptides after the restoration of SR due to atrial stunning [9, 12]. Furthermore, as a consequence of atrial stretch, the injection of contrast dye into the LAA may also induce the upregulation of BNP [13].

The LAA plays an essential role in maintaining cardiac homeostasis via the secretion of BNP. The implantation of the LAAC device may lead to consecutive LAA flow limitations and may alter the blood level of BNP. However, the level of BNP in patients treated with the hybrid procedure was not lower than that in the simple CA group. Luani et al. [14] observed no significant change in the level of NT-proBNP six months after LAAC. Grieshaber et al. [15] reported similar findings in patients undergoing left atrial appendage amputation. These results suggest that, even after losing part of the LAA function, the heart can still maintain liquid balance as an endocrine organ via secreting BNP. LAAC, as a concomitant procedure to CA, did not affect the endocrine function of the heart.

### 4.2. Mechanical Cardiac Function

The impact of the combined procedure and simple CA on the mechanical function of the heart was evaluated by conventional and two-dimensional STE. The analysis of *Ƹ* and SR using STE is a novel and precise way for functional assessment of LA [16]. Our results showed that LA Ƹ and SR improved in both groups after the procedure.

Continued improvement in mechanical cardiac function was observed in both groups. There was no significant difference in LAEF, SRe, and SRa (an indicator of LA conduit and pump function) between the two groups. However, the *Ƹ* and SRs (an indicator of LA reservoir function) were improved in patients who received CA alone, indicating that the addition of LAAC to CA may exert detrimental effects on LA reservoir function. In our study, we noticed some discrepancies in the outcome values in 2 chamber and 4 chamber views. The difference is mainly owing to the different measurement regions of the LA wall. The endocardial border of the LA wall was manually traced in apical 4- and 2-chamber views, thus delineating a region of interest composed of 6 segments (Figure 3). In the 4-chamber view, a region of the septal and lateral walls of LA were delineated. However, the anterior and posterior walls of the LA were traced in a 2-chamber view. Also, the reproducibility of LA-SRa in some patients was poor, which may have led to inconsistency between the two-chamber and four-chamber results.

LA reservoir function represents the ability of the LA to store pulmonary venous return during left ventricular contraction and isovolumetric relaxation. The LA conduit function represents the ability of the LA to passively transfer blood into the left ventricle. The LA contractive function shows the contraction ability of the LA during the last diastolic phase [17]. LAA acts as a reservoir of the left atrium and has been reported to be more compliant than the LA main chamber, which may explain the decline in LA reservoir function after adding LAAC to CA [18]. A reduced LA reservoir strain has been shown to correlate with LA wall fibrosis in AF patients. Some studies show that LA reservoir function is predictive of maintaining sinus rhythm in AF patients undergoing catheter ablation and is an independent predictor of LA reverse remodeling [19–21].

Our findings have potential implications for clinical practice and provide insights to optimize therapeutic strategies for AF patients. The LAA is a hormone-producing organ with contractile function, and the effects of LAAC on cardiac function remain controversial. Previous studies have demonstrated that percutaneous LAAC did not translate into any significant changes in LA function assessed by LA volume and strain indices [22, 23]. De Maat et al. [24] also reported the same results in patients with surgical LAA exclusion. However, Coisne et al. [25] found that LAAC was associated with an improvement in LA mechanical function through the Frank–Starling mechanism. The same result was observed in patients with left atrial appendage exclusion by epicardial ligation [26]. Thus, the best strategy for LAAC is still a topic of discussion. The combination therapy can confer benefit by performing only one surgical procedure [27]. Li et al. [28] reported that the combined procedure had beneficial effects on left atrial structural reverse remodeling. However, recent evidence indicates that a one-stop procedure may increase the risk of peridevice leakage and could be replaced with a staged hybrid procedure [29]. From the aspect of cardiac function, our results demonstrate that patients may benefit from the one-stop hybrid procedure combining catheter ablation and device occlusion of the LAA. As rhythm control plays a key role in improving cardiac function, it outweighs the detrimental effects of LAAC by enhancing LA reservoir function, and the additional LAAC procedure did not affect the improvement of endocrine and mechanical cardiac function after CA.

### 4.3. Study Limitations

The study was limited in several ways. First, the sample size was small. Secondly, the study cohort is relatively heterogeneous, including both SR and AF patients at baseline. Thirdly, this study is limited by its retrospective nature. Further prospective studies are needed to confirm the current findings.

## 5. Conclusion

The combined procedure of CA and LAAC significantly improved cardiac function in AF patients. Compared to simple CA, based on CA with LAAC intervention, it does not significantly change LA pressure and endocrine function, but may lead to a decline in the LA reservoir function.

## Figures and Tables

**Table 1:** Clinical characteristics of the study population at baseline.

	LAAC + CA (*n* = 62)	CA (*n* = 62)	*p* value
Age (years)	64.2 ± 8.3	62.5 ± 7.2	0.20
Male gender *n* (%)	32 (52)	37 (60)	0.37
Smoking *n* (%)	5 (8)	9 (15)	0.26
Alcohol *n* (%)	7 (11)	9 (15)	0.59
Abnormal INR *n* (%)	3 (5)	4 (6)	0.70
Hypertension *n* (%)	31 (50)	39 (63)	0.15
Coronary artery disease *n* (%)	26 (42)	18 (29)	0.13
Diabetes mellitus *n* (%)	9 (15)	9 (15)	1
Stroke *n* (%)	28 (45)	26 (42)	0.72
Bleeding *n* (%)	2 (3)	1 (2)	0.56
CHA2DS2- VASc score	3.8 ± 1.6	3.3 ± 1.7	0.10
HAS-BLED score	3 (1, 3)	2 (1, 3)	0.11
Sinus rhythm preoperative *n* (%)	20 (32)	22 (35)	0.70

*Drugs before ablation n (%)*			
AAD	20 (32)	24 (39)	0.45
Beta-blocker	28 (45)	32 (52)	0.47
ACEI or ARB	25 (40)	22 (35)	0.58
Aldosterone receptor antagonists	5 (8)	6 (10)	0.75
Other diuretics	10 (16)	8 (13)	0.61

The data are shown as the mean ± SD or *n* (%). LAAC, left atrial appendage closure; CA, catheter ablation; AF, atrial fibrillation; AAD, antiarrhythmic drugs; ACEI, angiotensin converting enzyme inhibitor; ARB, angiotensin-2 receptor blockade.

**Table 2:** Changes of mechanical cardiac functions after the combined procedure and simple catheter ablation.

	Baseline	1 week	1 month	3 months	*p* value
*LAAC combined with CA group (n* *=* *47)*					
LAEF	33.1 ± 14.1	37.8 ± 12.2	42.4 ± 12.7	44.8 ± 13.2	˂0.001
*Ƹ* (2-chamber)	15.6 ± 7.1	21.1 ± 9.8	22.8 ± 8.5	25.4 ± 11.1	˂0.001
*Ƹ* (4-chamber)	17.6 ± 9.2	21.3 ± 9.2	23.5 ± 8.4	24.9 ± 9.5	˂0.001
SRs (2-chamber)	0.96 ± 0.4	1.2 ± 0.5	1.2 ± 0.4	1.3 ± 0.6	0.002
SRs (4-chamber)	1.0 ± 0.5	1.2 ± 0.4	1.3 ± 0.4	1.3 ± 0.4	0.001
SRe (2-chamber)	−1.4 ± 0.5	−1.4 ± 0.6	−1.4 ± 0.5	−1.5 ± 0.6	0.041
SRe (4-chamber)	−1.4 ± 0.5	−1.5 ± 0.6	−1.5 ± 0.5	−1.6 ± 0.6	0.019
SRa (2-chamber)	—	−1.3 ± 0.6	−1.5 ± 0.6	−2.4 ± 1.1	0.195
SRa (4-chamber)	—	−1.3 ± 0.7	−1.5 ± 0.6	−1.6 ± 0.7	0.003

*Simple CA group (n* *=* *49)*					
LAEF	34.1 ± 17.3	41.3 ± 10.3	46.2 ± 10.9	48.9 ± 13.1	˂0.001
*Ƹ* (2-chamber)	19.0 ± 11.1	26.0 ± 8.1	30.4 ± 10.5	32.5 ± 12.7	˂0.001
*Ƹ* (4-chamber)	21.2 ± 10.9	26.3 ± 7.5	27.9 ± 7.7	30.4 ± 10.6	˂0.001
SRs (2-chamber)	1.1 ± 0.7	1.3 ± 0.4	1.5 ± 0.4	1.6 ± 0.6	˂0.001
SRs (4-chamber)	1.2 ± 0.8	1.4 ± 0.5	1.4 ± 0.4	1.6 ± 0.5	0.006
SRe (2-chamber)	−1.5 ± 0.8	−1.5 ± 0.5	−1.6 ± 0.9	−1.6 ± 0.8	0.23
SRe (4-chamber)	−1.7 ± 0.7	−1.6 ± 0.5	−1.6 ± 0.6	−1.8 ± 0.7	0.151
SRa (2-chamber)	—	−1.5 ± 0.6	−2.0 ± 0.8	−2.0 ± 1.2	0.001
SRa (4-chamber)	—	−1.3 ± 0.8	−1.6 ± 0.6	−1.9 ± 0.9	˂0.001

The data are shown as the mean ± SD. LAEF, left atrial ejection fraction; *Ƹ*, strain; SR, strain rate. SRs, strain rate during ventricular systole; SRe, strain rate during early ventricular diastole; SRa, strain rate during atrial systole.

**Table 3:** Periprocedural characteristics and postoperative complications.

	LAAC + CA (*n* = 62)	CA (*n* = 62)	*p* value
CA procedure time, min	105.7 ± 28.3	109.7 ± 22.8	0.39
LAAC procedure time, min	44.4 ± 15.9		
CA fluoroscopy time, min	6 (4, 7)	6 (5, 8.25)	0.33
LAAC fluoroscopy time, min	5 (3.75, 6)		

*Complication n %*			
Pericardial effusion required drainage	1 (2)	0 (0)	0.32
Bleeding	0 (0)	1 (2)	0.32
Stroke or transient ischemic attack	0 (0)	0 (0)	N/A
Vascular events	2 (3)	1 (2)	0.56

*TEE 3-months follow up*			
Device-related thrombus	1 (2)		
Residual flow (>5 mm)	0 (0)		

LAAC, left atrial appendage closure; CA, catheter ablation; TEE, transesophageal echocardiography.

## Data Availability

The datasets generated for this article are available from the corresponding author on reasonable request.

## Abbreviations

LAAC: Left atrial appendage closure
CA: Catheter ablation
AF: Atrial fibrillation
LA: Left atrial
BNP: Brain natriuretic peptide
SR: Strain rate
LAA: Left atrial appendage
NVAF: Nonvalvular atrial fibrillation
SR: Sinus rhythm.
